# Performance Assessment of Five Different Soil Moisture Sensors under Irrigated Field Conditions in Oklahoma

**DOI:** 10.3390/s18113786

**Published:** 2018-11-05

**Authors:** Sumon Datta, Saleh Taghvaeian, Tyson E. Ochsner, Daniel Moriasi, Prasanna Gowda, Jean L. Steiner

**Affiliations:** 1 Department of Biosystems and Agricultural Engineering, Oklahoma State University, Stillwater, OK 74078, USA; saleh.taghvaeian@okstate.edu; 2 Department of Plant and Soil Sciences, Oklahoma State University, Stillwater, OK 74078, USA; tyson.ochsner@okstate.edu; 3 USDA-ARS Grazinglands Research Laboratory, El Reno, OK 73036, USA; daniel.moriasi@ars.usda.gov (D.M.); prasanna.gowda@ars.usda.gov (P.G.); jean.steiner@ars.usda.gov (J.L.S.)

**Keywords:** volumetric water content, salinity, soil moisture depletion, irrigation management

## Abstract

Meeting the ever-increasing global food, feed, and fiber demands while conserving the quantity and quality of limited agricultural water resources and maintaining the sustainability of irrigated agriculture requires optimizing irrigation management using advanced technologies such as soil moisture sensors. In this study, the performance of five different soil moisture sensors was evaluated for their accuracy in two irrigated cropping systems, one each in central and southwest Oklahoma, with variable levels of soil salinity and clay content. With factory calibrations, three of the sensors had sufficient accuracies at the site with lower levels of salinity and clay, while none of them performed satisfactorily at the site with higher levels of salinity and clay. The study also investigated the performance of different approaches (laboratory, sensor-based, and the Rosetta model) to determine soil moisture thresholds required for irrigation scheduling, i.e., field capacity (FC) and wilting point (WP). The estimated FC and WP by the Rosetta model were closest to the laboratory-measured data using undisturbed soil cores, regardless of the type and number of input parameters used in the Rosetta model. The sensor-based method of ranking the readings resulted in overestimation of FC and WP. Finally, soil moisture depletion, a critical parameter in effective irrigation scheduling, was calculated by combining sensor readings and FC estimates. Ranking-based FC resulted in overestimation of soil moisture depletion, even for accurate sensors at the site with lower levels of salinity and clay.

## 1. Introduction

Irrigated agriculture, a major contributor to the United States (U.S.) economy, plays a vital role in supplying the demand for food, feed, and fiber. Although only 27% of all croplands in the U.S. are irrigated, this sector is responsible for nearly 50% of crop revenues [1]. Sustaining high levels of food production through irrigated agriculture requires large amounts of water. In 2010, irrigation was the second largest consumer of freshwater withdrawals in the U.S., accounting for approximately 33% (approximately 159 million m^3^ year^−1^) of the total water withdrawals [2]. Irrigation water sources, however, are usually limited in amount and are subject to increasing competition. In addition, more variability in precipitation patterns is expected due to climate change, which may threaten the availability of irrigation water supplies [3,4]. These challenges create the need to optimize irrigation management and avoid over- or under-irrigation. Over-irrigation, in addition to wasting water and valuable nutrients, can create favorable conditions for pests and diseases, increase energy costs, and reduce the lifespan of irrigation infrastructure. It can also result in erosion of topsoil and contamination of downstream water resources due to movement of water-soluble chemicals [5]. In contrast, under-irrigation reduces crop yield and negatively impacts economic viability of agricultural production.

Several advanced technologies are available to assist with achieving and implementing optimized irrigation management, including weather stations, air- and spaceborne remote sensing platforms, computer models, plant feedback sensors, and soil moisture sensors [6,7]. Soil moisture sensors, in particular, can be used effectively to improve irrigation management [8]. As a tool for irrigation scheduling, these sensors have been shown to increase crop yields while conserving water [8,9,10,11]. For example, Zotarelli et al. [12] showed that users who manage irrigation with soil moisture sensors applied 15 to 51% less irrigation water compared to fixed-time irrigation plan and observed a crop yield increase of 11 to 26% in Florida, U.S. In addition, sensors can provide continuous estimate of soil moisture conditions in a nondestructive way at a reasonable cost and usually require little maintenance over their lifetime [13]. Soil moisture sensors include tensiometers, neutron gauges, electromagnetic sensors, electrical resistance sensors, and heat dissipation sensors, to name a few [14]. Among these different types, electromagnetic sensors have been widely used by producers for irrigation scheduling.

Despite their numerous advantages, electromagnetic sensors are sensitive to soil salinity and clay content. The impact of soil salinity on sensor readings of soil volumetric water content (*θ_v_*) (m^3^ m^−3^) has been highlighted in several studies [15,16,17]. For example, Wyseure et al. [16] reported that *θ_v_* error was acceptable at soil bulk electricity conductivity (EC) (dS m^−1^) levels below 2.0 dS m^−1^, and Schwartz et al. [18] found that *θ_v_* estimates were not affected at bulk EC levels below 2.8 dS m^−1^. These thresholds are exceeded in many irrigated areas in arid/semi-arid regions, where there is a great need for improving irrigation management using sensor technologies. The results from prior studies on the impact of clay content have been somewhat variable. Rüdiger et al. [19] observed overestimation error in *θ_v_* that increased with clay content. In contrast, Fares et al. [20] observed underestimation of *θ_v_* for electromagnetic sensors due to high clay content, which was more prevalent at lower soil moisture content. Mittelbach, et al. [21] reported both under- and overestimation errors at different depths of a clay loam soil in Switzerland. In light of these variable results, and since high salinity and clay content conditions are encountered in many agricultural fields, there is a need to undertake further field studies to investigate the accuracy of electromagnetic soil moisture sensors under varying levels of salinity and clay content.

The goal of this study was to evaluate the performance of soil moisture sensors for irrigation scheduling purposes under low and high salinity/clay content conditions. Specific objectives were to (1) assess the performance of five different commercially available electromagnetic sensors in estimating *θ_v_* in situ under soils with variable salt and clay content, (2) compare the accuracy of several approaches of determining soil moisture thresholds used in irrigation scheduling, and (3) investigate the accuracy of estimated soil moisture depletion based on sensor readings and different threshold approaches.

## 2. Materials and Methods

### 2.1. Sensor Description

Five commercially available electromagnetic sensors were evaluated in this study: TDR315, CS655, GS1, SM100, and CropX.

#### 2.1.1. TDR315

The TDR315 (Acclima Inc., Meridian, ID, USA) is a recently commercialized sensor for agricultural applications [18]. This sensor operates on principles of Time Domain Reflectometry (TDR) that estimates the soil apparent permittivity (*K_a_*) (unitless) at relatively higher frequencies (3.5 GHz), which are less sensitive to bulk EC compared to lower frequency electromagnetic techniques [22]. Conventional TDR sensors have a problem sustaining high frequency signals because of signal attenuation in the sensor’s coaxial cables. The TDR315 addresses this issue by embedding all the electronics required for pulse generation and waveform acquisition in a compact circuit within the probe head. The data are transmitted digitally via SDI-12 (Serial Data Interface at 1200 baud) protocol, which is an asynchronous, ASCII, serial communications protocol and can support a cable length of up to 60 m. The sensor shares the same advantages of the conventional TDRs, but, it is more portable, affordable, and convenient to use [18]. These sensors have a planar three-conductor transmission line, each 15 cm long, and transmit the incident pulse in the center rod and two exterior grounds. The TDR315 reports volumetric water content (*θ_v_*) (%) based on a proprietary dielectric mixed model which estimates *K_a_* using Topp equation (Equation (1)) [17]. The sensor also reports soil temperature (°C), bulk relative permittivity (unitless), bulk EC (µS cm^−1^), and soil pore water EC (µS cm^−1^). DataSnap SDI-12 data-loggers from the same manufacturer were used with TDR315 sensors to collect data on hourly basis.

(1) $$\theta_{v}=4.3\times 10^{-6}\times (K_{a}{}^{3})-5.5\times 10^{-4}\times (K_{a}{}^{2})+2.92\times 10^{-2}\times (K_{a})-5.3\times 10^{-2}$$

#### 2.1.2. CS655

The CS655 sensor (Campbell Scientific, Inc., Logan, UT, USA) is a water content reflectometer. An electronic pulse is sent from the probe head and reflected at the end of the rods (12 cm in length). Upon detecting the returned pulse, another pulse is sent. Then, the probe records the frequency of these pulses and inverses the frequency as period in microseconds (µs). This period is impacted by the velocity of electromagnetic pulse, which is influenced by *K_a_* [17,23]. The probe estimates *θ_v_* from *K_a_* using the Topp equation (Equation (1)) [17]. Apart from *θ_v_*, other measured parameters include period average (µs), soil’s relative dielectric permittivity (unitless), bulk EC (dS m^−1^), and soil temperature (°C). Like the TDR315, the CS655 communicates with a data-logger using an SDI-12 interface. To collect hourly data, CR1000 data-loggers (Campbell Scientific, Inc., Logan, UT, USA) were used in this study.

#### 2.1.3. GS1

The GS1 sensor (METER Group, Inc., Pullman, WA, USA) estimates *θ_v_* by generating an electromagnetic field to measure the dielectric constant of the surrounding medium. This sensor uses capacitance and frequency domain technology and operates at 70 MHz [24]. It provides oscillating waves to the sensor rods that charge in response to the dielectric of the material. The sensor quantifies the charge and provides a raw value (RV) that is strongly correlated with *θ_v_* (Equation (2)). The GS1 has a rugged design and is capable of remaining in the soil for a long time. It has a two-rod design, with each rod measuring 5.5 cm in length. Hourly data were collected throughout the cropping season using EM5B analog data-loggers (METER Group, Inc., Pullman, WA, USA).

(2) $$\theta_{v}=3.62\times 10^{-4}(\mathrm{RV})-0.554$$

#### 2.1.4. SM100

The WaterScout SM100 sensor (Spectrum Technologies, Aurora, IL, USA) has two electrodes functioning as a capacitor, with surrounding soil acting as the dielectric. The capacitor is driven by an 80 MHz oscillator and converts the soil’s dielectric permittivity to an output signal, which correlates with *θ_v_*. Watchdog 1400 data-loggers from the same manufacturer were used to collect hourly data.

#### 2.1.5. CropX

The CropX sensor (CropX Ltd., Tel Aviv, Israel) integrates soil moisture sensing and a cellular communication package. The sensor electrodes are built into a helical wing attached to a central shaft for installation with reduced soil disturbance. The sensor measures soil moisture based on the amplitude domain reflectometry. When the amount of water changes in the soil, the sensor measures the change in amplitude differential due to changes in dielectric permittivity, which directly correlates to changes in water content. CropX is a multiprobe sensor that measures *θ_v_* at 20 and 46 cm depths in the soil.

### 2.2. Study Sites

**Figure 1:**
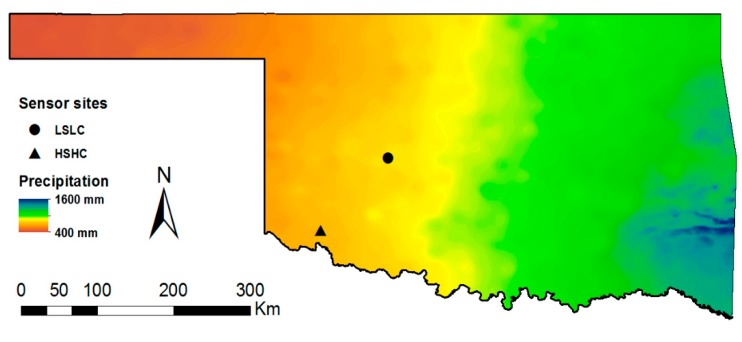
Experimental study site locations.

The study took place during the 2017 crop growing season. Two sites were selected for sensor installation, one with lower salinity and lower clay content (LSLC) located in central Oklahoma and the other in southwest Oklahoma with higher salinity and higher clay content (HSHC). Figure 1 shows the location of the study sites overlaid on the map of long-term mean annual precipitation across Oklahoma, obtained from Daly et al. [25]. The LSLC site had a Pond Creek fine sandy loam soil (fine-silty, mixed, superactive, thermic Pachic Argiustolls) while the HSHC site had a Hollister silty clay loam soil (fine, smectitic, thermic Typic Haplusterts). The EC of the soil solution (1:1 soil–water ratio) was 1.2 dS m^−1^ at LSLC compared to 7.0 dS m^−1^ at HSHC. Table 1 provides additional information on soil characteristics at each site. In addition to variations in soils, the two sites were different in crop types, irrigation systems, and climatic conditions. Corn (*Zea mays* L.) was planted at the LSLC site under a center-pivot irrigation system, while the HSHC site was under furrow-irrigated cotton (*Gossypium hirsutum* L.). Key meteorological parameters for each site are given in Table 2.

### 2.3. Experimental Setup

Four replications of TDR315, CS655, GS1, and SM100 and two replications of CropX were installed on 7/20/2017 and 7/27/2017 at LSLC and HSHC sites, respectively. The sensors were used with manufacturer-provided data-loggers and calibrations because the results obtained in this manner would best represent the conditions that irrigators and farm managers would face in the field [26]. Therefore, the raw *θ_v_* readings reported by the sensors were used in analysis without any alteration [13]. It should be noted that developing and utilizing site-specific calibrations can significantly improve accuracies if the required technical and financial resources are available to users. All sensors were installed at a depth of 20 cm from the soil surface. The top 20 cm is important for plant water uptake as root distribution of plants is denser in this layer than deeper in the soil profile [27].

At each replication, a pit was dug between two rows of crops to install the soil moisture sensors. Physical properties of soil in each pit were determined in the Soil Physics Laboratory at Oklahoma State University (OSU) by taking undisturbed soil cores (diameter = 2.5 cm, length = 5.1 cm) using the Sample Ring Kit (Model C, Eijkelkamp Soil & Water, Inc., Giesbeek, The Netherlands) on the day of sensor installation. Soil textural information (particle size distribution) were determined by hydrometer following the protocol proposed by Ashworth, et al. [28]. Additionally, four replications of soil samples were taken at each site on the installation day to measure soil salinity. The salinity test was done by Soil, Water and Forage Analytical Laboratory at OSU using the 1:1 soil water extraction method [29].

Sensors were inserted horizontally into the side wall of the pit (undisturbed soil) so that the rods of the sensors were on top of each other (vertical orientation) and the middle point of the sensor rods was directly under the crop row. The *θ_v_* readings are often impacted by the sensor installation procedure [30], so extra care was taken to maintain minimal disturbance to the surrounding soil while inserting the rods. The spacing between the sensors was determined based on the volume of influence of individual sensors plus an additional distance to eliminate any possible interference. This spacing between the sensors were varied from 10 to 18 cm depending on the volume of influence of sensors’ electromagnetic field. Then, wires were run below and away from the sensors for some distance to avoid creating any preferential flow channels. After that, the wires were run through PVC pipes to the data-logger encasement. The CropX sensors were installed using the spiral auger provided by the manufacturer to minimize soil disturbance.

The excavated soil was collected in different buckets for different soil layers and carefully used to backfill the pits, attempting to recreate the original bulk density. Precipitation amounts were recorded by a tipping bucket rain gage (model TE525-L, Campbell Scientific, Inc., Logan, UT, USA) at the LSLC site, whereas, these measurements were collected from an Oklahoma Mesonet weather station located 678 m to the southwest of the sensor installation location at the HSHC site [31]. Gravimetric soil samples (diameter = 3 cm, height = 5.1 cm) were collected using a Giddings soil sampling probe (Giddings Machine Company, Windsor, CO, USA) to estimate reference *θ_v_* (*θ_ref_*) (m^3^ m^−3^) throughout the crop growing season. On each sampling date, four gravimetric samples were taken at each site and the probe was centered at the sensor installation depth (20 cm). If there was an irrigation and/or precipitation event around the sampling dates, extra care was taken not to compact the areas above the sensors. Soil samples were put in plastic bags immediately after sample collection and kept out of sunlight to minimize evaporation. All soil samples of known volumes were oven-dried at 105 °C for 24 h and used to determine bulk density.

### 2.4. Soil Moisture Thresholds

Efficient irrigation management requires knowledge of two important soil moisture thresholds that indicate water availability for plant consumption [5]. These thresholds are field capacity (FC) and wilting point (WP). The FC is often estimated as the water retained at a soil matric potential of −33 kPa, although research has shown that this can result in underestimation of FC and −10 kPa may provide a more suitable approximation [32]. The WP is often estimated as the water retained at −1500 kPa [33]. These values can be different depending on soil texture, crop type, and other factors.

In this study, FC and WP were determined using three different approaches: laboratory, sensor-based, and the Rosetta model [34]. Undisturbed soil cores extracted from each site were used in laboratory tests where FC was determined at −33 kPa using the Tempe cell method and WP at −1500 kPa using the pressure plate method [35]. The sensor-based approach was based on ranking of the collected data following the procedure proposed in Hunt et al. [36]. This method uses sensor readings to estimate FC and WP as the 95th and the 5th percentiles of all *θ_v_* values collected during the study period. This method assumes that the hydrologic conditions during the measurement period result in *θ_v_* values, which span from values lower than WP to values higher than FC. The Rosetta model uses hierarchical pedotransfer functions to estimate van Genuchten water retention parameters [34]. In this study, three different FC-WP outputs were generated from the Rosetta model by providing different types and combination of input data. The three types of input data included (i) only the textural class of soils at study sites, (ii) textural information (percentages of sand, silt, and clay), and, (iii) textural information and bulk density. Estimated FC and WP from all methods described above were compared with those reported in the U.S. Department of Agriculture’s Web Soil Survey at each study site [37]. In addition to FC and WP, the available water content (AWC), which is the difference between FC and WP, was calculated and compared with values obtained from different methods described above [38].

To optimize irrigation management based on soil moisture sensing, sensor readings must be converted to soil moisture depletion (SMD) (m^3^ m^−3^). In this study, SMD was calculated as the difference between FC and *θ_v_*:

(3) $$SMD_{\left(i\right)}=\theta_{FC}-\theta_{v(i)}$$

where, *SMD*_(*i*)_ is the soil moisture depletion at the *i*th time-step, *θ_FC_* is the *θ_v_* at FC (constant) (m^3^ m^−3^), and *θ_v_*_(*i*)_ is the *θ_v_* at the *i*th time-step. In estimating SMD, *θ_v_*_(*i*)_ values were obtained from sensor readings and *θ_FC_* values were based on two different approaches, resulting in two SMD estimates for each sensor at each site. The two *θ_FC_* approaches were the laboratory and the ranking methods explained above. The results were compared against SMD estimates based on *θ_ref_* (gravimetric measurements) and laboratory *θ_FC_*. After SMD is estimated, it can be multiplied by the root zone depth to obtain an estimate of irrigation requirement in units of water depth.

### 2.5. Statistical Analysis

To evaluate the performance of the selected sensors, *θ_v_* readings of sensors were compared with *θ_ref_* values. Four statistical parameters, namely root mean square error (RMSE), RMSE-observations standard deviation ratio (RSR), mean bias error (MBE), and index of agreement (*k*) were estimated according to the following equations.

(4) $$\mathrm{RMSE}=\sqrt{\frac{1}{n}\sum_{i=1}^{n}{\left(P_{i}-O_{i}\right)}^{2}}$$

(5) $$\mathrm{RSR}=\frac{\mathrm{RMSE}}{{\mathrm{STDEV}}_{O_{\left(i\right)}}}=\frac{\sqrt{\sum_{i=1}^{n}{\left(O_{i}-P_{i}\right)}^{2}}}{\sqrt{\sum_{i=1}^{n}{\left(O_{i}-\bar{O}\right)}^{2}}}$$

(6) $$\mathrm{MBE}=\frac{1}{n}\sum_{i=1}^{n}\left(P_{i}-O_{i}\right)$$

(7) $$k=1-\left[\frac{\sum_{i=1}^{n}{\left(P_{i}-O_{i}\right)}^{2}}{\sum_{i=1}^{n}{\left(\left|P_{i}-\bar{O}\right|+\left|O_{i}-\bar{O}\right|\right)}^{2}}\right]$$

where, *n* is the sample size, *i* is the index of sample pairs, *P* is the sensor reading (predicted), *O* is the *θ_ref_* (observed), and $\bar{O}$ is the mean of all *θ_ref_* values.

The accuracy categories outlined in Fares et al. [20] were adopted in this study for interpreting RMSE values. These categories include good (RMSE ≤ 0.01 m^3^ m^−3^), fair (0.01 ≤ RMSE ≤ 0.05 m^3^ m^−3^), poor (0.05 ≤ RMSE ≤ 0.10 m^3^ m^−3^), and very poor (RMSE ≥ 0.10 m^3^ m^−3^). The RSR provides benefits of incorporating error index statistics and it includes a normalization factor applicable to various constituents [39]. The RSR varies from a value of zero indicating zero RMSE and a perfect model simulation to a large positive value. The performance of a model is determined by different categories of RSR: very good model fit (0.00 ≤ RSR ≤ 0.50), good model fit (0.50 ≤ RSR ≤ 0.60), satisfactory model fit (0.60 ≤ RSR ≤ 0.70), and unsatisfactory model fit (RSR > 0.70). However, these categories are based on simulations running on a monthly time-step. Moriasi et al. [39] noted that the acceptable range of RSR would increase in magnitude when using smaller time-steps, which was the case in this study. The MBE measures the average difference between sensor-estimated *θ_v_* and *θ_ref_*. A MBE of zero indicates the predicted and observed values are unbiased. A positive value of MBE means sensor is overestimating *θ_v_*, and negative MBE indicates underestimation [40]. The index of agreement (*k*) was used to determine how well the sensor-estimated *θ_v_* agreed with *θ_ref_* [41]. The value of *k* can range from zero to one, with one representing the highest level of agreement and zero representing complete disagreement [42].

In addition to the above statistical parameters, Pearson correlation coefficients (*r*) were calculated for pairwise sensor comparisons to evaluate the similarity in their temporal variations throughout the study period. Closely correlated temporal patterns have a *r* value near one, while this parameter is near zero in case of uncorrelated patterns [43]. Finally, linear regression models were fitted to sensor-estimated *θ_v_* and *θ_ref_* using the Minitab statistical software (version 17.3) (Minitab, Inc., State College, PA, USA) [44]. These linear models and the reported intercepts and slopes for each sensor can be used as field calibration equations in future applications at the study sites.

## 3. Results and Discussion

### 3.1. Sensor Performance

**Figure 2:**
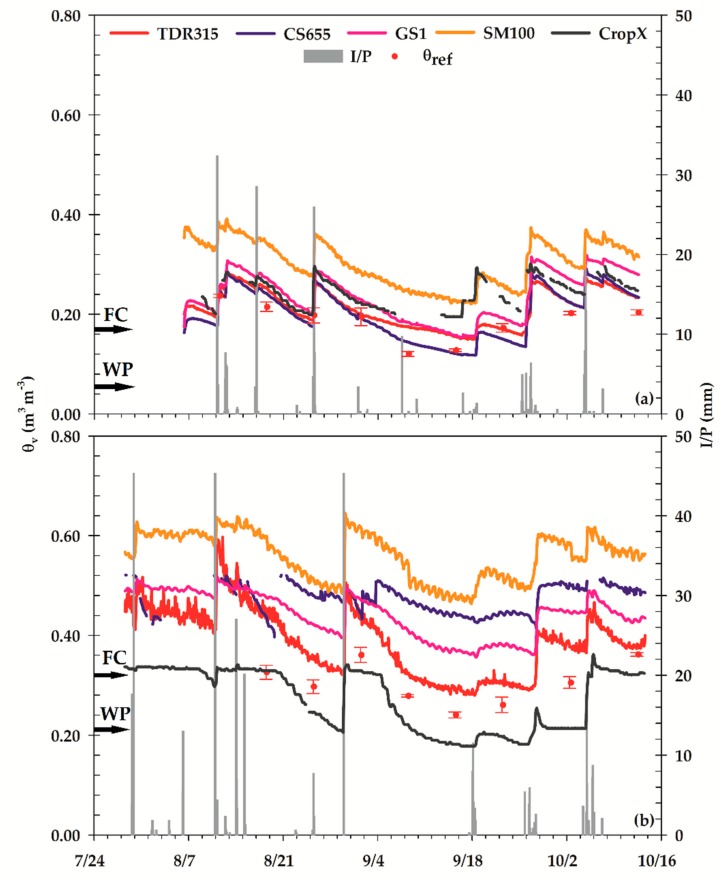
Time series of sensor-estimated *θ_v_* along with point measurements of *θ_ref_* at (**a**) lower salinity and lower clay content (LSLC) and (**b**) higher salinity and higher clay content (HSHC) sites. Error bars for *θ_ref_* represent standard error of mean. The FC and WP limits were determined in the laboratory.

The fluctuations in *θ_v_* were similar across all sensors at both study sites (Figure 2). All sensors responded to most irrigation and precipitation events. In some cases, there was little or no change in *θ_v_* following a watering event, mainly because the amount of water received was not large enough to reach sensor installation depth. The results of performance evaluation (statistical indicators) are summarized in Table 3. In general, all sensors performed better at the LSLC. At this site, the RMSE was the lowest for CS655 (0.019 m^3^ m^−3^), followed by TDR315 (0.028 m^3^ m^−3^) and GS1 (0.048 m^3^ m^−3^). These values belong to the fair accuracy category defined in Fares et al. [20], suggesting that CS655, TDR315, and GS1 can be implemented for effective irrigation scheduling under conditions similar to those of LSLC. The RMSE values obtained in this study were smaller than the RMSE values of 0.105 and 0.049 m^3^ m^−3^ reported by Singh et al. [45] for the CS655 and TDR315 in a loam soil, respectively. Adeyemi et al. [46] found a similar RMSE of 0.020 m^3^ m^−3^ for TDR315 and 0.050 m^3^ m^−3^ for GS1 in a sandy loam soil under laboratory conditions. The RMSE of CropX was 0.051 m^3^ m^−3^, which is in the poor category. The SM100’s RMSE was very poor (0.110 m^3^ m^−3^).

**Figure 3:**
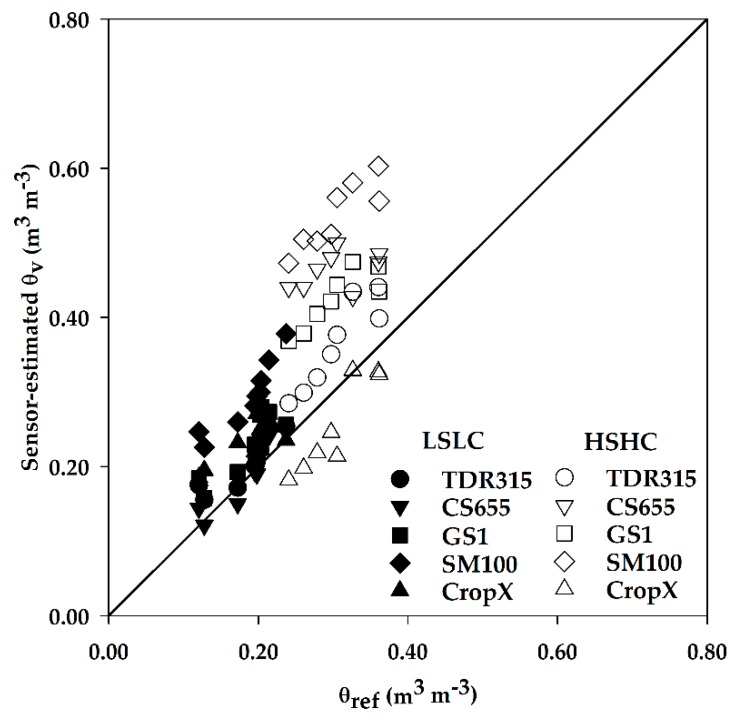
Sensor-estimated *θ_v_* vs. *θ_ref_* at LSLC and HSHC sites.

The MBE and RSR revealed similar patterns in sensor performance at the LSLC, with the CS655 performing the best, followed by TDR315, GS1, CropX, and SM100. The MBE indicated that all sensors overestimated *θ_v_* at LSLC. This overestimation can also be observed in Figure 3 as most of the points were above the 1:1 line. Overestimation of *θ_v_* by CS655 was observed by Kisekka et al. [47] and Michel et al. [48] too. Adeyemi et al. [46] found that TDR315 and GS1 underestimated *θ_v_* in sandy loam soil, but, with increasing clay content, the underestimation became overestimation. The RSR ranged from 0.53 for CS655 to 3.00 for SM100 at LSLC site. According to categories defined by Moriasi et al. [39], the CS655 had a good model fit whereas all other sensors were classified as having unsatisfactory model fit. But as mentioned previously, running a model on temporal resolution higher than monthly would warrant less strict performance rating. Therefore, higher RSR values are expected in this study because of hourly time-step analysis. This trend was also observed in a study by Wyatt et al. [49], which produced high RSR values at daily time-step.

All sensors had larger RMSE at the HSHC site compared to LSLC (Table 3). However, the magnitude of the increase in RMSE was not uniform and changed from a slight increase for CropX to over an eight-fold increase for CS655. The CropX sensor had the smallest RMSE, followed by TDR315, GS1, CS655, and SM100. The values of RMSE belonged to the poor accuracy category in case of CropX and TDR315 and very poor category for other sensors according to classifications in Fares et al. [20], suggesting that none of the sensors can be implemented for effective irrigation scheduling under conditions similar to those of HSHC. In addition, the variability of readings among the replications of the same sensors increased at HSHC; the average standard deviation (SD) ranged from 0.021 m^3^ m^−3^ for TDR315 to 0.050 m^3^ m^−3^ for CS655. At LSLC, the average SD varied from 0.011 m^3^ m^−3^ for TDR315 to 0.023 m^3^ m^−3^ for GS1. The average SD of *θ_ref_* was 0.015 m^3^ m^−3^ at LSLC and 0.010 m^3^ m^−3^ at HSHC.

High clay content and elevated levels of salinity seem to be the main reasons behind lower sensor accuracies at the HSHC site. Adeyemi et al. [46] concluded that the errors in TDR315 and GS1 would increase with an increase in soil salinity level. In addition, Wyseure et al. [16] reported that the error in TDR sensors would remain within reasonable limits if the bulk EC is kept less than 2 dS m^−1^. The bulk EC at HSHC, however, was well over this threshold. The MBE estimates were larger at HSHC than LSLC and showed that all sensors except CropX overestimated *θ_v_*. This is also evident in Figure 3. Most of previous studies have reported overestimation error for TDR sensors under saline conditions. This is mainly due to the fact that in saline soils, the dielectric permittivity measured by TDR increases and therefore *θ_v_* is overestimated as mentioned in Dalton [15]. However, Schwartz et al. [18] found that TDR315 underestimated *θ_v_* in a saline Pullman clay loam soil. The RSR values followed a pattern similar to other error indicators at HSHC, having the smallest value of 1.34 for CropX and the largest value of 5.66 for SM100.

Some noise in *θ_v_* readings of the TDR315 at HSHC can be seen in Figure 2b. Schwartz et al. [18] reported that TDR315 sensors were insensitive to bulk EC up to 2.8 dS m^−1^ and corresponding pore water EC up to 7.3 dS m^−1^. The bulk EC and pore water EC exceeded these thresholds at HSHC on many days at the beginning of the study period. This might have caused signal attenuation that induced noise in *θ_v_* readings. This noise was quantified using standard deviation (SD) in *θ_v_* among the replications. At the beginning of the growing season, the SD had a range of zero to 0.099 m^3^ m^−3^ and an average of 0.021 m^3^ m^−3^ at HSHC for TDR315, which is much larger when compared to the range of 0.002 to 0.043 m^3^ m^−3^ and average of 0.012 m^3^ m^−3^ at LSLC during the same period. The observed noise was reduced later in the growing season, probably due to decrease in soil EC because of leaching of salts by irrigation water.

**Figure 4:**
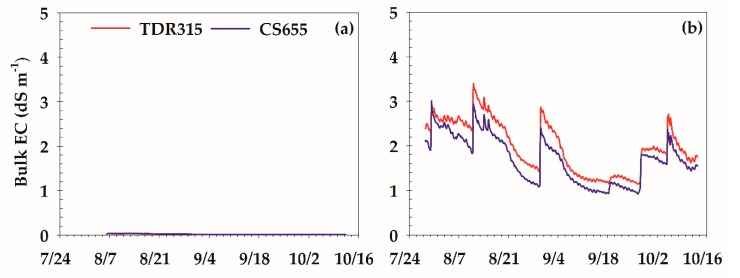
Time series of sensor-estimated Bulk electricity conductivity (EC) at (**a**) LSLC and (**b**) HSHC sites.

The hourly bulk EC estimates from TDR315 and CS655 were in agreement with soil EC determined in the laboratory and showed the significant difference between the two study sites (Figure 4). Both sensors reported small bulk EC at LSLC with similar ranges of 0.1 to 0.4 dS m^−1^. At HSHC, however, the bulk EC was significantly larger with ranges of 1.1 to 3.4 and 0.9 to 3.0 dS m^−1^ based on TDR315 and CS655 sensors, respectively. 

In utilizing soil moisture sensors for irrigation management, obtaining a complete time series is as important as taking accurate readings. In this study, CropX and CS655 had significant data gaps for different reasons. On average, 41% of the CropX data were missing at LSLC compared to less than one percent at HSHC. Several correspondences with the manufacturer revealed that the potential reason behind this issue could be the tall corn canopy at LSLC, which can block the transmitted signals. Upon recommendation from the manufacturer, extension antennas were installed on CropX sensors at LSLC. The observed crop height was 2.16 m and the extension antennas were installed in such a way that the tops of the antennae were 1.91 m from the ground. However, this modification did not help with the apparent transmission problem.

The CS655 had 21% missing data at HSHC. Sugita et al. [50] conducted a reliability test on CS655 and found that the sensor was missing 64% of the measurements when exposed to high salinity levels (bulk EC = 1.2–2.1 dS m^−1^). The bulk EC at HSHC was larger than the values reported in Sugita et al. [50]. In addition to high salinity, the HSHC site had relatively high clay content (38.7%). The clay particles have highly charged surface areas which increase dielectric losses and cause the apparent permittivity (*K_a_*) values to go outside the acceptable range of Topp equation [17]. The combined effect of higher soil salinity and clay content results in the attenuation of the electromagnetic signal from the sensor [18].Therefore, the sensor fails to report *θ_v_* in case of *K_a_ ≥* 42 and *θ_v_* ≥ 0.52 m^3^ m^−3^ as the internal logical test rejects these data.

Linear regression equations were developed to estimate *θ_ref_* based on sensor-estimated *θ_v_* (Table 4). These equations can be used to get more accurate *θ_v_* readings in areas matching this study’s local conditions. At LSLC, the regression models were all statistically significant at α = 0.05, with *r^2^* values ranging from 0.57 for CropX to 0.85 for CS655. Although SM100 had low accuracy, the high *r^2^* value (0.84) indicates that this sensor had high degree of correlation with the reference values. At the HSHC site, the linear regression model for CS655 was not statistically significant. Models of other sensors were significant and had *r^2^* values varying from 0.73 to 0.85.

### 3.2. Correlations between Sensors

In general, the Pearson’s correlation coefficients (*r*) of *θ_v_* readings were larger at LSLC than HSHC (Table 5). At this site, the strongest correlation (*r* = 0.99) was between TDR315 and CS655 and the weakest was between CropX and SM100 (*r* = 0.79). The correlation coefficients for CropX were smallest among all sensors at the LSLC site, ranging from 0.79 to 0.81. Despite being the least accurate sensor, SM100 had strong correlation with the top two accurate sensors, i.e., TDR315 and CS655. This indicates that SM100 closely followed the temporal changes in *θ_v_* of more accurate sensors. At HSHC, the correlation between TDR315 and GS1 was the strongest (*r* = 0.97). The SM100 also had strong correlations with TDR315, GS1, and CropX. On the other hand, CS655 had weak correlations with other sensors.

The strong correlation between sensors with different accuracies suggests that the response of less accurate sensors to soil moisture fluctuations was similar to those of more accurate sensors. The differences in *θ_v_* readings were relatively constant over the study period (offset error). This provides an opportunity for potential utilization of less accurate sensors in some limited applications where the user is only interested in determining the movement of the water front in the soil profile. One example of this application is leaching salts below the root zone. In this case, the user needs to ensure water front has moved below the bottom of the root zone. Another example is preventing deep percolation to ensure applied water remains within the root zone and that soluble chemicals are not transported to shallow groundwater resources.

### 3.3. Soil Moisture Thresholds

At LSLC, the FC and WP estimated in the laboratory were similar to the output of the Rosetta model based on textural class, textural information, and textural information plus bulk density (Table 6). Thresholds obtained from USDA’s Web Soil Survey (USDA-WSS) were slightly larger than the results of the laboratory and Rosetta methods. However, the estimates based on the ranking of sensor readings were significantly larger than those of the other methods. The FC and WP values were larger at HSHC compared to LSLC irrespective of the method used because of larger clay content in the soils. The FC values from the Rosetta model and the USDA-WSS were either similar or slightly smaller than those obtained with the laboratory approach. All ranking estimates of FC were significantly larger than those with laboratory approach except for CropX, which was slightly larger. In the case of WP, estimates from the Rosetta model were significantly smaller than those with the laboratory approach, while USDA-WSS reported a similar value. Ranking method estimates were significantly larger except for CropX. The differences between AWC estimates of the ranking and laboratory methods were smaller than the differences in the FC and WP estimates of the same methods at both sites, mainly because overestimations in FC and WP estimates of the ranking method were of similar magnitudes and thus cancelled out to a large extent.

Results of this study reveal that the Rosetta model is capable of accurately estimating soil moisture thresholds even with minimal input data (textural classes). The USDA-WSS also performed satisfactorily, despite the fact that it is based on coarse soil surveys. However, the ranking method resulted in significant overestimation of FC when compared to laboratory estimates, ranging from 59 to 117% at the LSLC and from 6 to 94% at HSHC site. The difference between WP estimates of the ranking and laboratory methods varied from 100 to 283% at LSLC and from −14 to 129% at HSHC. A potential reason behind this poor performance could be that the full range of soil moisture conditions was not experienced at both sites during the period of study. However, this situation could be the case in many irrigated areas, since producers attempt to replenish soil moisture well before it reaches WP to avoid water stress and yield loss. Another reason behind the poor performance of the ranking method is the error in sensor readings, especially at HSHC, where most sensors overestimated soil moisture due to high clay content and elevated salinity levels.

**Figure 5:**
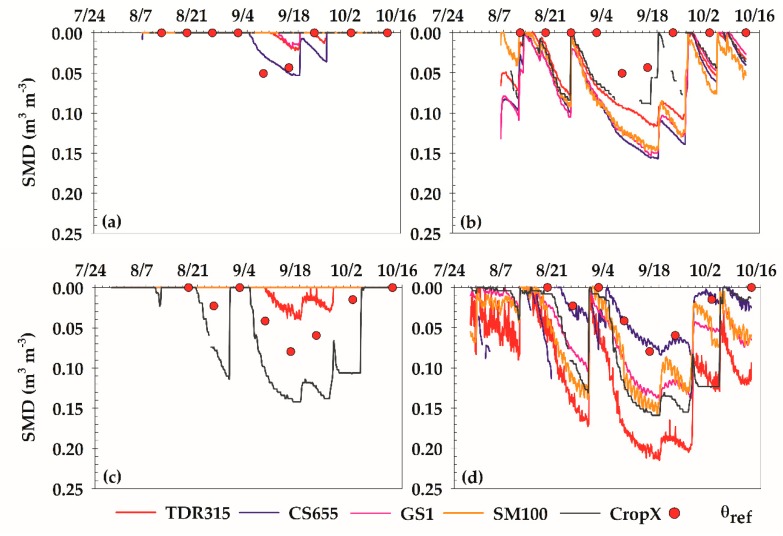
Time series of hourly soil moisture depletion (SMD) estimated based on sensor readings of *θ_v_* and FC estimates from laboratory (**a**) and ranking (**b**) methods at LSLC site and laboratory (**c**) and ranking (**d**) methods at HSHC site. Dots represent SMD estimated based on *θ_ref_* and FC estimates from laboratory method.

Variations in hourly SMD are presented in Figure 5. In this figure, dots represent observed SMD based on *θ_ref_* and laboratory-determined FC, while lines represent sensor SMD based on sensor *θ_v_* and FC from two methods: laboratory and ranking. The Rosetta model was not considered here because the FC values obtained from the model were similar to those of the laboratory. At LSLC, observed SMD values were zero except on two sampling dates in early September. This is because this site was under full to slightly over-irrigation at most times during the study period. The only exception for the same period was in September when crop water demand outpaced irrigation application. Possible underestimation of *θ_FC_* in the laboratory method may have contributed to zero SMD on most measurement dates too. In this study, a soil matric potential of −33 kPa was used to measure *θ_FC_*. But as mentioned before, this value can be as high as −10 kPa in sandy loam soil, resulting in a larger *θ_FC_* and consequently a larger SMD estimate. Sensor SMDs based on laboratory-FC had similar patterns, indicating no depletion during the study period except in the month of September (Figure 5a). On the other hand, sensor SMDs based on ranking-FC showed significant depletions at most times, reaching values as large as 0.15 m^3^ m^−3^ (Figure 5b). This increase in SMD is mainly due to overestimation of FC in the ranking method, since the same sensors readings were used in both SMD approaches.

At the HSHC site, the observed SMD indicated a larger depletion, especially during early September to early October. This pattern was expected since this site was under a low-frequency (7–10 days) flood irrigation regime that was not able to meet cotton water demand during the hot and dry month of September. At this site, sensor SMDs based on laboratory-FC showed no depletion except for CropX and TDR315. The SMD estimates of CropX were larger and the SMD estimates of TDR315 were smaller than observed SMD. This is because CropX underestimated *θ_v_*, while TDR315 overestimated this parameter. The overestimation errors of the other sensors were so large that their *θ_v_* readings were above laboratory-FC at all times, resulting in no depletion. The sensor SMDs based on ranking-FC were significantly larger than those based on laboratory-FC, except for CS655. This was because of the overestimation of FC by the ranking method. Hence, depletion was calculated at most times. The SMDs of CS655 were similar to the observed SMD, since the overestimation errors in *θ_v_* readings and ranking-FC were similar in magnitude.

## 4. Conclusions

The performance of five types of commercially available soil moisture sensors was evaluated at two fields with significantly different salinity levels and clay contents. The sensors included TDR315, CS655, GS1, SM100, and CropX. The accuracy of each sensor was determined by comparing its readings with gravimetric measurements of soil water content obtained at several times during the study period. In general, all sensors responded to wetting and drying events. The TDR315, CS655, and GS1 sensors had acceptable accuracies for managing irrigations at the site with low salinity and low clay content (LSLC) based on root mean square error (RMSE). However, none of the sensors performed satisfactorily at the site with high salinity and high clay content (HSHC), with RMSE estimates that were up to eight times larger compared to the values at LSLC. In addition, high levels of noise were observed in TDR315 due to high salinity and out-of-range responses and consequently missing readings in case of CS655 sensor. A potential solution for using soil moisture sensors in irrigation scheduling under such conditions is the use of site-specific calibrations.

For practical irrigation scheduling, sensor readings must be used in conjunction with soil moisture thresholds of field capacity (FC) and wilting point (WP) in order to estimate soil moisture depletion (SMD) and consequently irrigation requirement. In this study, FC and WP values determined in the laboratory using undisturbed soil cores were compared against those obtained from three independent approaches: the Rosetta model, the ranking of sensor readings, and the values reported in the U.S. Department of Agriculture’s Web Soil Survey (USDA-WSS). The Rosetta model was capable of providing estimates similar to those of the laboratory approach, regardless of the type and number of input data used in the model. The USDA-WSS approach resulted in acceptable estimates of FC and WP. The ranking method, however, significantly overestimated FC and WP at both sites, even for accurate sensors. The ranking method did not perform well in estimating SMD either, except for one sensor at the HSHC site where the overestimation error in FC was similar to overestimation error in soil water content and canceled each other out. The results of this study show that two major conditions are required before the ranking method can be used effectively in estimating soil moisture thresholds: sensor readings that are the basis of calculations must be accurate; and, the full range of moisture conditions from below WP to above FC must be experienced during the data collection period.

This study contributes to the existing knowledge on sensor-based irrigation scheduling through quantifying the accuracies of five widely-used soil moisture sensors as impacted by soil clay content and salinity, as well as investigating the effectiveness of different soil moisture threshold estimation approaches for agricultural irrigation applications. The results highlight the wide range of accuracies that exist among soil moisture sensors and methods for determining soil moisture thresholds. Such a wide range creates major challenges in utilizing soil moisture sensors for irrigation scheduling applications. As new sensors are being developed frequently, studies like this need to be conducted under variable field conditions to evaluate the performance of the new sensors and to provide guidelines on how they can be used for irrigation scheduling purposes.

## Figures and Tables

**Table 1:** Soil properties at study sites.

Site	Soil Texture	Particle Size Distribution			EC ^¥^	*θ_v_* (m^3^ m^−3^)			K_sat_^†^
		% Sand	% Silt	% Clay	(dS m^−1^)	Sat. ^‡^	FC ^§^	WP *	(mm day^−1^)
LSLC	Fine sandy loam	72.2	14.4	13.4	1.2	0.34	0.17	0.05	390.0
HSHC	Silty clay loam	23.5	37.8	38.7	7.0	0.39	0.32	0.21	32.4

^¥^ Electrical conductivity. ^‡^ Saturation level; ^§^ Field capacity at −33 kPa; * Wilting point at −1500 kPa; ^†^ Saturated hydraulic conductivity.

**Table 2:** Twenty-year (1997–2016) average annual and study period (July to October 2017) meteorological parameters obtained from Oklahoma Mesonet weather network.

Parameter	Annual		Study Period	
	LSLC	HSHC	LSLC	HSHC
Total Prec. ^1^ (mm)	752	616	451	340
Mean R_s_^2^ (MJ m^−2^)	17.1	17.7	19.9	21.8
Minimum T_air_^3^ (°C)	9.4	10.0	18.1	18.9
Maximum T_air_ (°C)	22.1	24.1	30.6	31.2
Mean T_air_ (°C)	15.4	16.8	23.9	24.8
Minimum RH ^4^ (%)	41.9	37.6	45.5	44.7
Mean VPD ^5^ (kPa)	0.9	1.0	1.0	1.1
Mean U_2_^6^ (m s^−1^)	2.5	2.5	3.0	2.5

^1^ Precipitation; ^2^ Daily accumulation of solar radiation; ^3^ Daily air temperature; ^4^ Daily relative humidity. ^5^ Daily vapor pressure deficit; ^6^ Daily wind speed at 2.0 m above the ground.

**Table 3:** Performance indicators of soil moisture sensors.

Indicators	TDR315		CS655		GS1		SM100		CropX	
	LSLC	HSHC	LSLC	HSHC	LSLC	HSHC	LSLC	HSHC	LSLC	HSHC
RMSE (m^3^ m^−3^)	0.028	0.064	0.019	0.165	0.048	0.122	0.110	0.233	0.051	0.055
RSR	0.76	1.55	0.53	3.99	1.31	2.97	3.00	5.66	2.53	1.34
MBE (m^3^ m^−3^)	0.020	0.053	0.008	0.160	0.042	0.121	0.108	0.233	0.045	−0.049
k	0.85	0.69	0.94	0.30	0.69	0.41	0.44	0.26	0.58	0.75

**Table 4:** Parameters and the *p*-values of the linear regression equation: *θ_ref_* = Slope × (sensor *θ_v_*) + Intercept.

Site	Sensor	Intercept	Slope	*r* ^2^	*p*-Value
LSLC	TDR315	−0.017	0.975	0.80	0.001
	CS655	0.036	0.771	0.85	<0.001
	GS1	0.017	0.737	0.70	0.005
	SM100	−0.033	0.747	0.84	0.001
	CropX	−0.052	1.030	0.57	0.018
HSHC	TDR315	0.056	0.683	0.85	0.001
	CS655	−0.056	0.774	0.20	0.267 ^Ŧ^
	GS1	−0.108	0.971	0.73	0.007
	SM100	−0.165	0.873	0.79	0.003
	CropX	0.137	0.656	0.85	0.001

^Ŧ^ The linear regression model was not statistically significant.

**Table 5:** Pearson correlation coefficients among installed sensors at study sites.

**LSLC**					
	**TDR315**	**CS655**	**GS1**	**SM100**	**CropX**
TDR315	1.00				
CS655	0.99	1.00			
GS1	0.97	0.99	1.00		
SM100	0.95	0.95	0.92	1.00	
CropX	0.79	0.81	0.81	0.79	1.00
**HSHC**					
	**TDR315**	**CS655**	**GS1**	**SM100**	**CropX**
TDR315	1.00				
CS655	0.50	1.00			
GS1	0.97	0.57	1.00		
SM100	0.90	0.48	0.90	1.00	
CropX	0.86	0.42	0.85	0.78	1.00

Note all correlation coefficients were significant at *p* = 0.05.

**Table 6:** Estimates of field capacity (FC), wilting point (WP), and available water content (AWC) (all in m^3^ m^−3^) obtained from various methods.

Method	LSLC			HSHC		
	FC	WP	AWC	FC	WP	AWC
Laboratory ^1^	0.17	0.06	0.11	0.32	0.21	0.09
Rank-TDR315 ^2^	0.27	0.16	0.11	0.49	0.29	0.20
Rank-CS655 ^2^	0.27	0.12	0.15	0.51	0.43	0.08
Rank-GS1 ^2^	0.31	0.16	0.15	0.50	0.37	0.13
Rank-SM100 ^2^	0.37	0.23	0.14	0.62	0.48	0.14
Rank-CropX ^2^	0.28	0.17	0.11	0.34	0.18	0.16
Rosetta-TC ^3^	0.17	0.06	0.11	0.31	0.12	0.19
Rosetta-TI ^4^	0.17	0.07	0.10	0.29	0.14	0.15
Rosetta-TBD ^5^	0.15	0.07	0.08	0.26	0.14	0.12
USDA-WSS ^6^	0.21	0.12	0.09	0.29	0.21	0.08

^1^ Laboratory measurement; ^2^ Ranking method performed for each sensor; ^3^ Rosetta model using soil textural class only; ^4^ Rosetta model using soil textural information (% sand, silt, and clay); ^5^ Rosetta model using textural information and bulk density; ^6^ USDA’s Web Soil Survey.
